# Magnetic particle spectroscopy for Eu-VSOP quantification in intestinal inflammation: distinguishing nanoparticle signals from dietary contamination

**DOI:** 10.1039/d5na00452g

**Published:** 2025-08-28

**Authors:** Norbert Löwa, Laura Golusda, Daniela Paclik, Heike Traub, Mathias Schannor, Jessica Saatz, Christian Freise, Matthias Taupitz, Britta Siegmund, Anja A. Kühl, Frank Wiekhorst

**Affiliations:** a Physikalisch-Technische Bundesanstalt (PTB), Working Group 8.23 Metrology for Magnetic Nanoparticles Abbestr. 2-12 10587 Berlin Germany norbert.loewa@ptb.de; b Charité – Universitätsmedizin Berlin, Corporate Member of Freie Universität Berlin and Humboldt, Universität zu Berlin, Department of Gastroenterology, Infectious Diseases and Rheumatology Campus Benjamin Franklin, Hindenburgdamm 30 12200 Berlin Germany; c Charité – Universitätsmedizin Berlin, Corporate Member of Freie Universität Berlin and Humboldt, Universität zu Berlin, iPATH.Berlin Campus Benjamin Franklin, Hindenburgdamm 30 12200 Berlin Germany; d Bundesanstalt für Materialforschung und -prüfung (BAM), Division 1.1 Inorganic Trace Analysis Richard-Willstätter-Str. 11 12489 Berlin Germany; e Charité – Universitätsmedizin Berlin, Corporate Member of Freie Universität Berlin and Humboldt, Universität zu Berlin, Department of Radiology-Experimental Radiology Campus Mitte, Virchowweg 11 10117 Berlin Germany; f Charité – Universitätsmedizin Berlin, Corporate Member of Freie Universität Berlin and Humboldt, Universität zu Berlin, Department of Radiology Campus Benjamin Franklin, Hindenburgdamm 30 12200 Berlin Germany

## Abstract

Magnetic nanoparticles are gaining increasing attention as a promising alternative to gadolinium-based contrast agents in magnetic resonance imaging, primarily due to their low toxicity. In this study, we investigated the use of magnetic iron oxide nanoparticles in mouse models of intestinal inflammation to assess their potential for detecting changes in the extracellular matrix. For magnetic quantification, we employed magnetic particle spectroscopy, which offers high sensitivity and minimal interference from biological tissue. However, we observed significant variations in magnetic signals within the intestine, as well as measurable signals in control animals, indicating possible magnetic contamination. By doping the nanoparticles with europium, we were able to confirm this suspicion through quantitative elemental analysis. Examination of mouse feed and feces allowed us to identify the source of contamination. Based on these findings, we developed a method to reliably distinguish genuine signals of magnetic nanoparticles from those caused by external magnetic contaminations. This approach is essential to ensure reliable results in future diagnostic and preclinical research.

## Abstract 2

Magnetic nanoparticles show great potential for detecting intestinal inflammation, with magnetic particle spectroscopy enabling reliable distinction of true nanoparticle signals from external contamination.

## Graphical abstract

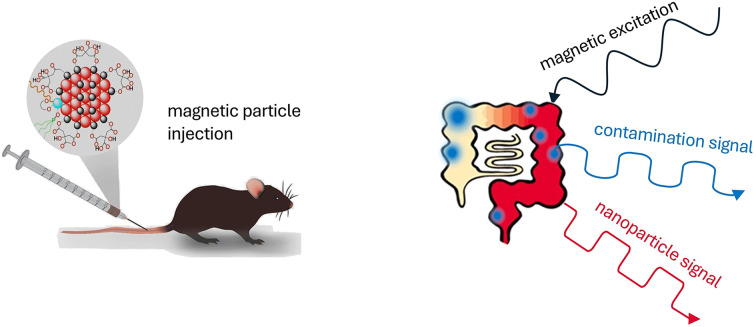

## 1. Introduction

Magnetic nanoparticles (MNP) are becoming increasingly important in preclinical molecular and cellular imaging, particularly in magnetic resonance imaging (MRI), due to their superior properties compared to gadolinium-based contrast agents. MRI is a vital tool for soft tissue analysis and is widely used in clinical practice due to its non-invasive nature, lack of ionizing radiation, and high spatial resolution. Gadolinium-based contrast agents have long been the gold standard for MRI contrast agents, significantly enhancing diagnostic accuracy. However, their limited use in patients with renal insufficiency and growing concerns over potential toxicity^1,2^ – intensified by confirmed brain deposition even with an intact blood–brain barrier and normal renal function – underscore the urgent need for safer and more effective alternatives such as MNP. Pre-clinical studies have already shown the biocompatibility and low toxicity of ferrimagnetic iron oxide-based contrast agents.^3–7^ Besides using MNP as a contrast agent in MRI, the emerging technique of magnetic particle imaging (MPI) represents an imaging modality that directly visualizes the distribution of MNP in real time. Thus, MPI employs MNP as a tracer and does not involve ionizing radiation, making it a promising alternative for medical imaging applications.

MNP consist of a magnetically active core coated with biocompatible stabilizers such as dextran, citrate, silica, or polyethylene glycols to prevent particle clustering, enhance stability in biological environments, and enable functionalization for the targeted delivery to specific tissues or cells.^8^ A special class of MNP are very small superparamagnetic iron oxide particles (VSOP), with a diameter less than 10 nm.^9^ Research has shown that VSOP have the potential to target inflammation sites in atherosclerotic lesions and inflamed endothelial cells in the brain.^10,11^ Their binding and uptake are influenced by components of the extracellular matrix (ECM).^12^ The question arises whether VSOP can target inflammations in other organs with an altered ECM. It is known that inflammatory bowel diseases, such as ulcerative colitis (UC) and Crohn's disease (CD), cause changes in the ECM. The binding of VSOP to altered ECM and their detection (*e.g.*, *via* MRI and MPI) could aid in the diagnosis and early detection of these intestinal inflammations.

Europium-doped VSOP (Eu-VSOP) facilitate straightforward detection of nanoparticle distribution in tissues using fluorescence microscopy^13^ and laser ablation inductively coupled plasma mass spectrometry (LA-ICP-MS).^14–16^ This modification enables the specific localization and quantification of Eu-VSOP, since it is not possible to differentiate Fe from VSOP and endogenous iron using *e.g.* ICP-MS detection. The background levels of Eu in biological samples (*e.g.*, tissue, feces, and mouse nutrition) are very low, so that Eu is a suitable label for the presence of VSOP. Although acute toxicity from Eu is rare, the long-term effects of its accumulation, particularly from repeated exposure in medical applications, are not well understood. This lack of data on chronic toxicity introduces uncertainty to its safety profile, making non-doped Eu-VSOP the preferred option for future clinical use.

Accurately quantifying Eu-VSOP in tissue is crucial for assessing their diagnostic potential. However, this task is complicated by the risk of magnetic contamination, which can skew results. Magnetic contamination can occur during sample collection and processing, especially in clinical settings where instruments and storage conditions may introduce magnetic particles. Additionally, external environmental factors such as airborne particulate matter, dust, or residues from cleaning agents can contribute to magnetic contamination. These contaminants can affect the accuracy of magnetic nanoparticle-based diagnostics or therapeutic applications, as they can lead to false readings and misinterpretations. Therefore, strict control measures, including sterile handling techniques and careful monitoring of environmental conditions, are essential to minimize the risk of contamination and ensure reliable results. For example, in the intestinal tract, which is constantly exposed to exogenous material like food, contamination is particularly challenging to avoid.^17,18^ This makes a reliable quantification of VSOP difficult.

For magnetic quantification, various techniques are available,^19–22^ among which Magnetic Particle Spectroscopy (MPS) stands out due to its high specificity, sensitivity, and quantitative accuracy, while effectively eliminating background interference from biological tissues. MPS enables non-invasive, real-time detection of magnetic nanoparticles without requiring complex sample preparation, making it ideal for both clinical diagnostics and research applications. Additionally, MPS is based on the same physical principles as MPI, facilitating the translation of MNP research to imaging applications. MPS detects the nonlinear magnetic response of MNP, allowing for quantifying Eu-VSOP in tissues such as the intestine. However, MPS can be sensitive to ferromagnetic contamination as well, which may interfere with results. Nevertheless, MPS offers a distinct advantage: the signal from each type of MNP, including Eu-VSOP, exhibits a unique signature. This enables the potential distinction between signals from MNP like Eu-VSOP and magnetic contaminants, ensuring accurate quantification.

In this study, we aimed to detect and quantify Eu-VSOP in mouse models of intestinal inflammation. Intravenous injections of Eu-VSOP were given to both healthy and colitic mice, with healthy animals serving as baseline controls. Sham controls were also included, where mice were injected with PBS. Rag1 ko mice served as baseline controls for the mouse model of transfer colitis, while C5BL/6 wildtype mice served as baseline controls for the model of DSS-induced colitis. However, we observed a high variability of iron mass fractions in intestinal tissues, which could suggest contamination with ferro- or ferrimagnetic iron. Therefore, we investigated the source of contamination by analyzing mouse nutrition and feces. Based on our findings, we propose a method to distinguish between MPS signals coming from the Eu-VSOP and those originating from magnetic contamination.

## 2. Results and discussion

### 2.1 Magnetic particle spectroscopy as a method to quantify and distinguish MNP types

#### 2.1.1 Harmonic amplitude and phase analysis

**Fig. 1:**
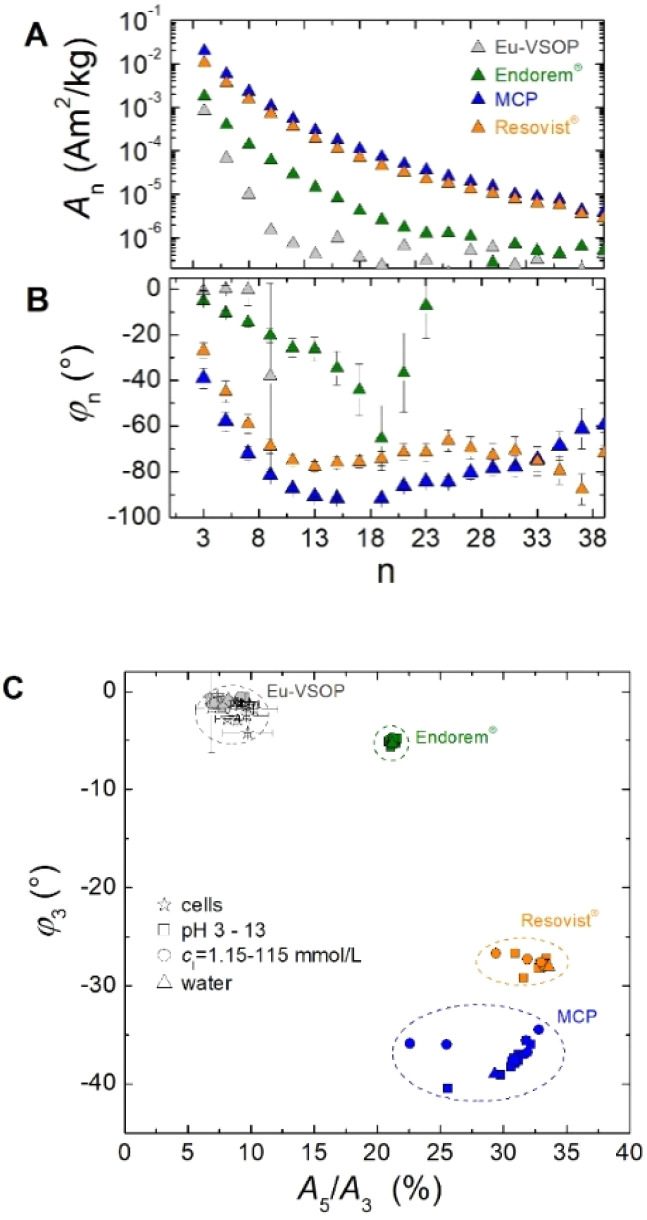
MPS measurements of different MNP types (VSOP, Endorem®, Resovist®, MCP) reveal distinct signal characteristics in both the amplitude (*A*_n_) (A) and phase spectrum (*ϕ*_n_) (B). A two-dimensional plot of the phase (*ϕ*_3_) and amplitude ratio (*A*_5_/*A*_3_) further enables clear differentiation between MNP types (C). While environmental factors such as cellular uptake, pH variations, and ionic strength influence the magnetic response, the different MNP types remain clearly distinguishable by MPS. The dashed circles indicate the regions where the signals of the different MNP types are located and serve as visual guides only.

First, we consider the harmonic amplitude *A*_n_ and phase *φ*_n_ of different MNP types as measured by MPS. The results are displayed in Fig. 1A. For consistent comparison the moments *A*_n_ are normalized to the iron amount of the samples.

The amplitudes of the harmonics *A*_n_ exhibit the characteristic decline for all MNP samples, though with different shapes (Fig. 1A). Eu-VSOP exhibit a steep drop in amplitude spectra, attributed to the smaller magnetic moments because of the smaller particle size. In contrast, MCP and Resovist® maintain relatively flat harmonic spectra, indicative of larger magnetic moments and high responsiveness to high-frequency excitation, likely linked to larger particle sizes and lower magnetic anisotropy. Endorem® exhibits an intermediate behavior, with its amplitude spectrum between those of Eu-VSOP and MCP/Resovist®.

The phase angles (*ϕ*_n_) exhibit distinct behavior for each sample as well (Fig. 1B). Eu-VSOP possess a low phase angle of approximately −2° for *n* = 3 to *n* = 9, while harmonics *n* > 9 are below the noise threshold. In contrast, Endorem®, MCP, and Resovist® display a larger phase lag declining as harmonic order increases. The lower and more negative phase values (larger phase lag between the oscillating excitation field and response of the moments) in these samples suggest differences in magnetic anisotropy and relaxation mechanisms compared to Eu-VSOP.

The distinct amplitude and phase signals highlight the capability of MPS to accurately identify and distinguish between different MNP types. Moreover, the MPS signal amplitude is directly proportional to the iron content in the sample, making it a reliable tool for quantification of MNP in tissue samples with unknown concentrations. A key prerequisite for accurate quantification is that the dynamic magnetic behavior of the MNP in the tissue remains unchanged. To verify this, the harmonic ratio (*A*_5_/*A*_3_) and phase signal (*ϕ*_n_) can be utilized, as they are independent of MNP concentration and serve as robust indicators of consistency. The verification of the amplitude signal linearity (*A*_n_) and stability of the signals (*A*_5_/*A*_3_ and *ϕ*_n_) at dilution is shown for Eu-VSOP in Section S1 of the SI.

#### 2.1.2 Harmonic ratio *vs.* phase signal

Next, the ratio of the 5th to 3rd harmonic amplitude (*A*_5_/*A*_3_) was plotted against the phase of the third harmonic (*φ*_3_) (Fig. 1C), serving as a visual tool for identifying and differentiating MNP types. Eu-VSOP samples, characterized by its small particle size, appear in the upper left of the plot with a low harmonic ratio (*A*_5_/*A*_3_) of approximately 10% and a phase angle near 0°, *i.e.* the smaller magnetic moments follow the excitation with less delay. In contrast, MCP and Resovist®, systems with larger particle sizes, exhibit slower relaxation dynamics and are therefore visualized in MPS as clusters with higher harmonic ratios (*A*_5_/*A*_3_) at about 30% and more negative phase values of approximately −30°. Endorem® with a harmonic ratio of about 20% and a phase angle near −10° is found in between, highlighting its distinct magnetic signature.

The observed variations in harmonic ratio and phase angle reflect the unique relaxation dynamics and magnetic anisotropy of each nanoparticle type, which safely can be distinguished by MPS.

#### 2.1.3 Influence of environmental factors on MPS signals

Beyond the inherent magnetic properties of the MNP, Fig. 1C illustrates the influence of environmental factors on MPS signals, including variations in pH (3–13), ionic concentrations (*c*_i_ = 1.15–115 mmol L^−1^), and MNP uptake by cells. While MPS signals exhibit some sensitivity to these external conditions – reflected in shifts in harmonic ratios and phase angles – the characteristic fingerprints of each MNP type remain distinguishable. This highlights the ability of MPS to reliably differentiate between MNP types despite signal variations caused by environmental variations.

Overall, these findings confirm that MPS signals serve as unique fingerprints for distinguishing MNP samples. Importantly, this capability is preserved across different environmental conditions, underscoring the robustness of MPS as a diagnostic and analytical tool for biomedical and environmental applications.

### 2.2 Food as a source of contamination in intestinal tissues

#### 2.2.1 Variation of Eu-VSOP amount in intestinal tissues

**Fig. 2:**
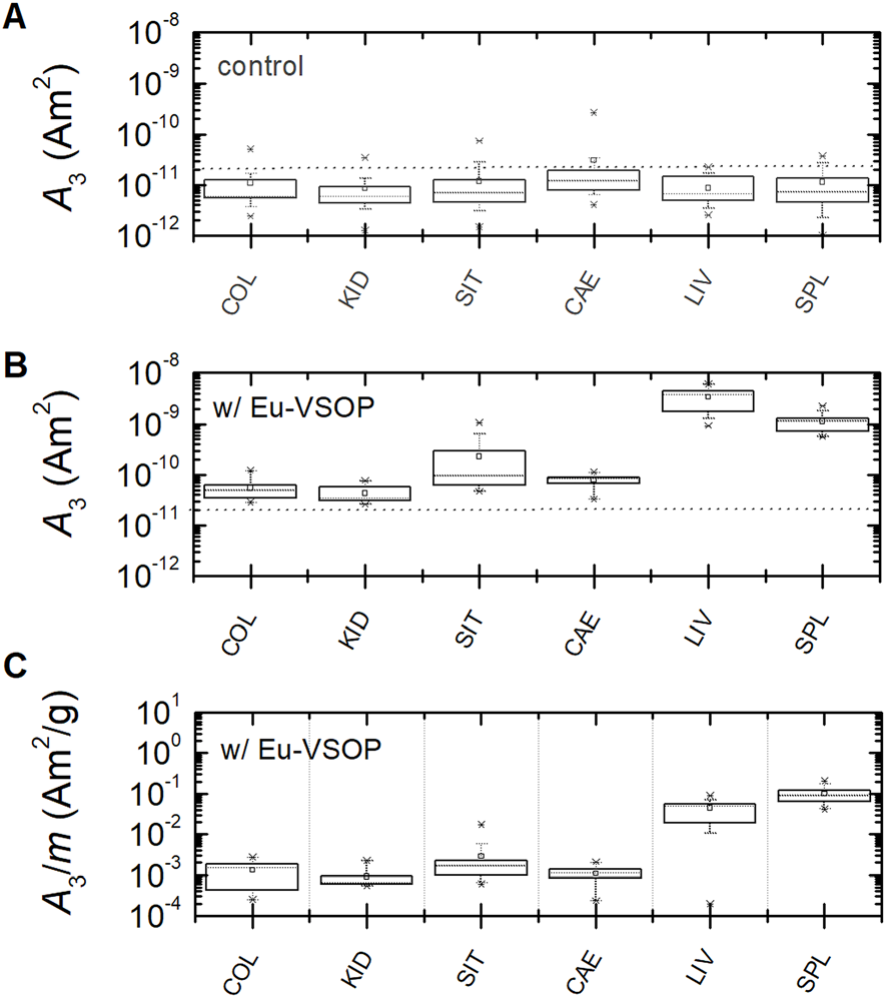
Box plots of MPS signal amplitude (*A*_3_) displaying the lower and upper quartiles, median (dotted line), mean (square), as well as standard deviation (whiskers), and outliers (asterisk). *A*_3_ values are shown for the colon (COL), kidney (KID), small intestine (SIT), caecum (CAE), liver (LIV) and spleen (SPL) in mice injected with PBS (A), as well as Eu-VSOP (B), and with normalization to tissue mass (C). The dotted grey lines in A and B denote the limit of detection (LOD = 2 × 10^−11^ A m2) of the third harmonic *A*_3_ see Section 4.2. Each box plot is based on eight independent measurements per group. The boxes represent the interquartile range (25th to 75th percentile), the whiskers indicate the 5th and 95th percentiles, and both the mean and median are shown. Outliers are displayed as individual points.

The magnetic iron content of Eu-VSOP in various tissue samples was quantified using the Eu-VSOP-specific amplitude 
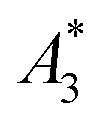
 (in units A m^2^ kg^−1^ (Fe)) of the MPS signal, as *A*_3_ is directly proportional to the magnetic iron amount (see Section 4.2). MPS measurements were performed on tissue samples from animals injected with Eu-VSOP as well as from control animals injected with PBS (without VSOP). In PBS-treated mice, the mean and median signal amplitude (*A*_3_) remained below the detection limit for all organs (Fig. 2A). In contrast, all animals that received Eu-VSOP intravenously exhibited increased *A*_3_ values in all organs, with the liver and spleen showing the highest signal intensities (Fig. 2B and C). The predominant accumulation in the liver is due to its role in the mononuclear phagocyte system, where Kupffer cells actively uptake nanoparticles from the bloodstream. Similarly, in the spleen, a secondary clearance organ, nanoparticles are sequestered by splenic macrophages and filtered through the sinusoids of the red pulp.

Interestingly, some organs of the control mice (Fig. 2A) displayed outliers above the MPS detection limit, suggesting the presence of ferri- or ferromagnetic iron. In particular, the intestinal tissue showed particularly high signal amplitudes, with the highest values detected in the caecum, probably indicating tissue contamination. Signal spectra *A*_n_ and *ϕ*_n_ from selected tissue samples are displayed in the supplementary material (Fig. S2).

#### 2.2.2 Magnetic contamination in feces and food

**Fig. 3:**
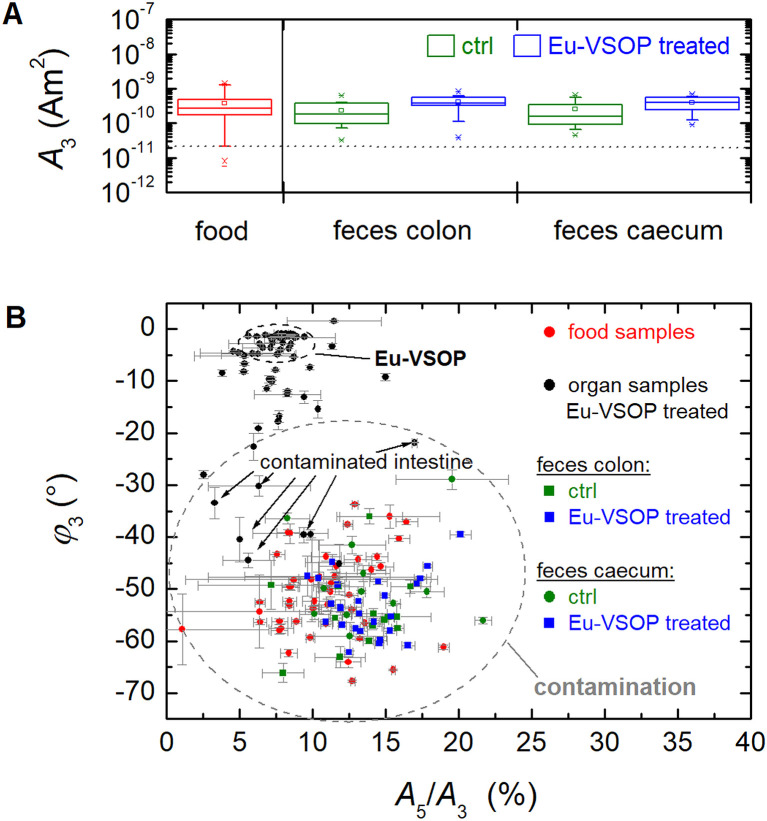
The MPS signal amplitude *A*_3_ of the feces isolated from colon and caecum from animals after injection of Eu-VSOP (blue) or PBS as a control (green) with higher signals in cecal than colonic samples (A). The dotted grey line denotes the limit of detection (LOD = 2 × 10^−11^ A m^2^) of the third harmonic amplitude *A*_3_ (see Section 4.2). The two-dimensional plot (*A*_5_/*A*_3_, *ϕ*_3_) shows the magnetic signature of the food (red), the feces isolated from Eu-VSOP-treated animals (blue), and PBS-treated controls (green) as well as the organs from Eu-VSOP-treated animals (black) (B).

Since intestinal tissues from mice exhibited significantly high MPS signals even without Eu-VSOP injections, feces were isolated from the colon and caecum and analyzed using MPS. Signals from feces in both regions were well above the detection limit (LOD), with higher values observed in feces from the caecum. Notably, MPS signals were detected in all fecal samples, regardless of Eu-VSOP injection (Fig. 3A).

To determine the source of iron contamination in feces, the diet was analyzed using MPS. Despite high variability, food samples showed amplitude signals *A*_3_ as high as feces samples (Fig. 3A). Additionally, the spectral profiles of signals from feces and food were similar, strongly indicating that the diet is the primary source of magnetic contamination (Fig. S2A in the SI). As ingested food is digested, these magnetic contaminants travel through the gastrointestinal tract, become partially absorbed by intestinal tissue, and ultimately appear in the feces. As the retention time of feces in the caecum is higher than in the colon, the magnetic contamination is obviously higher in these tissue samples.

Unlike other methods for detecting ferro- or ferrimagnetic iron, MPS enables a clear distinction between dietary magnetic contamination and Eu-VSOP signals (Fig. 3B) by plotting the phase *ϕ*_3_ against the amplitude ratio *A*_5_/*A*_3_. In this representation, samples span a much broader range of *ϕ*_3_(*A*_5_/*A*_3_) values compared to Eu-VSOP systems shown previously (Fig. 1C). A specific range of *ϕ*_3_(*A*_5_/*A*_3_) values – corresponding to the Eu-VSOP variations in Fig. 1C and highlighted by the ellipsoid in the upper left of Fig. 3B – is assigned to Eu-VSOP (primarily organ samples from Eu-VSOP treated animals). All other samples, falling outside this range, are considered contaminated with MPS-visible magnetic material, exhibiting a non-linear dynamic magnetic response above the LOD (exclusively intestinal organ samples). Consequently, several tissue samples from animals not treated with Eu-VSOP (indicated by black arrows in Fig. 3B) are classified as contaminated based on their *ϕ*_3_ (*A*_5_/*A*_3_) values. Thus, spectral shape analysis using *ϕ*_3_ (*A*_5_/*A*_3_) enables MPS to exclude contaminating iron, ensuring accurate Eu-VSOP detection.

**Fig. 4:**
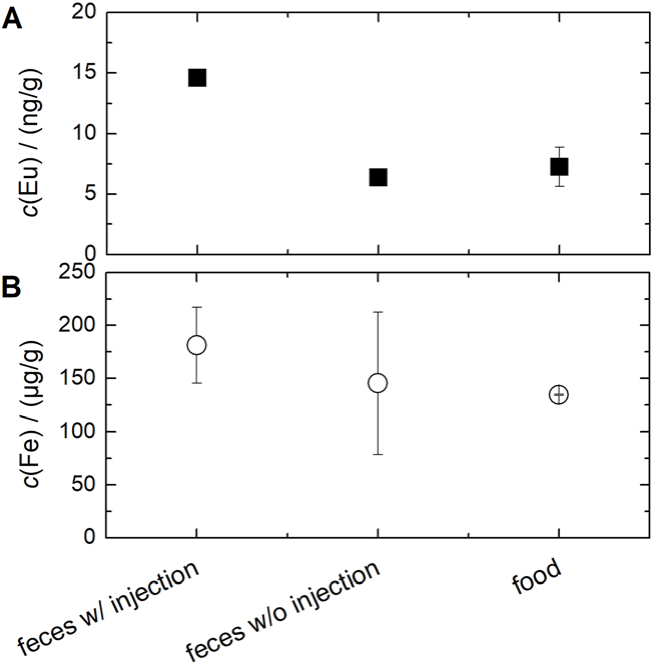
Europium (A) and iron contents (B) quantified by ICP-MS (Eu in ng g^−1^) or ICP-OES (Fe in μg g^−1^) in food samples (*n* = 2) and feces caecum samples from animals with and without Eu-VSOP injection (*n* = 3 each). While europium levels in the control group are comparable to those in the food sample, the Eu concentrations in the Eu-VSOP group are significantly higher.

Europium and iron contents in individual food and fecal samples were further quantified using inductively coupled plasma mass spectrometry (ICP-MS) or ICP optical emission spectrometry (ICP-OES) (Fig. 4). Feces caecum samples were analysed from animals with and without Eu-VSOP injection. The ICP-MS and ICP-OES results reflect the total Eu or Fe contents in the samples, as it is not possible to distinguish the source after sample digestion. The results confirmed that both food and fecal samples contained significantly high iron levels. The Fe content in the feces samples from animals with Eu-VSOP injection is on average slightly higher than in the samples from control animals and in the mouse feed. However, the Fe introduced by the Eu-VSOP injection accounts for only a small proportion of the total Fe content in the feces caecum samples. In addition, the Fe contents in the feces samples vary significantly, which could indicate differences between the animals, *e.g.* regarding the amount of feed consumed.

In contrast, the europium content in Eu-VSOP-treated animals is about twice as high as in the control group and food sample (Fig. 4A). This indicates that the Eu contents in the feces caecum samples of the control animals are probably due to the diet. These findings further support the previous MPS results.

#### 2.2.3 Elemental mapping of tissue samples by LA-ICP-MS

Elemental mapping using laser ablation in combination with an ICP time-of-flight mass spectrometer (LA-ICP-TOFMS) was performed as an additional method to obtain information on the spatial distribution of Eu and Fe. Liver and intestinal tissue thin sections from animals with transfer colitis with and without Eu-VSOP injection were analysed. By doping the iron oxide-based VSOP with europium (Eu-VSOP), a specific detection *via* LA-ICP-MS was enabled since it is not possible to distinguish Fe from VSOP from *e.g.* endogenous iron or iron contaminations due to sample preparation using ICP-MS detection.

**Fig. 5:**
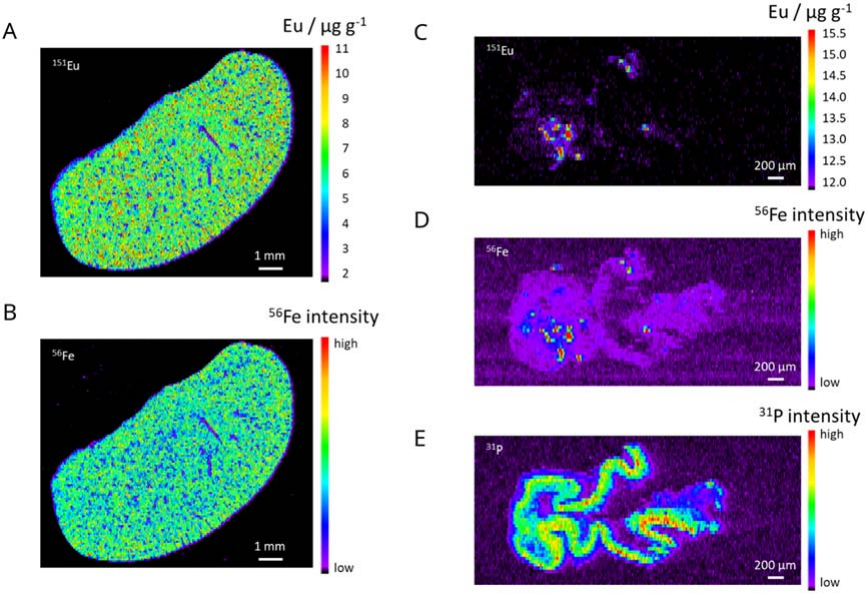
Elemental maps of liver and intestine thin sections from a mouse with transfer colitis after Eu-VSOP injection (animal #71) measured with LA-ICP-TOFMS. (A) and (B) Distribution of Eu and Fe in the liver, (C)–(E) distribution of Eu, Fe and P in the intestine. For Eu quantitative elemental maps are shown. The intensity distributions of ^31^P and ^56^Fe are shown for P and Fe respectively. P is used as a marker for the shape of the intestine sample. Liver: pixel size 5 μm × 80 μm, scale bar 1 mm; intestine: pixel size 2.5 μm × 40 μm, scale bar 200 μm.

**Fig. 6:**
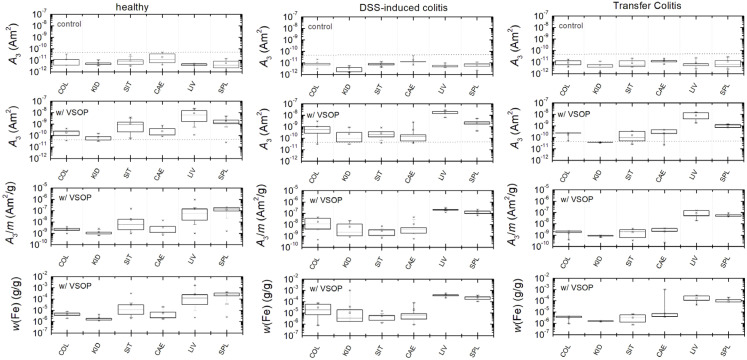
Corrected MPS quantification by excluding contaminated samples based on the magnetic signature analysis. The dotted grey lines in the A3 moment panels show the limit of detection (LOD) of the third harmonic *A*_3_ as explained in Section 4.2.

As expected, Eu was detected in liver and intestinal tissues from animals that had received the Eu-VSOP injection (Fig. 5). In the control animal (without Eu-VSOP injection) Eu could not be detected in the liver or intestine (data not shown). Similarly, as anticipated, iron was found in all liver samples, regardless of Eu-VSOP injection, since natural iron is primarily stored in the liver as ferritin, which is only paramagnetic, with ICP-MS detection not allowing differentiation. In addition, Fe was detected in the intestine of the animal after VSOP administration (Fig. 5D) as well as in the control animal (Fig. S3 in the SI). A comparison of the maps for Eu and Fe shows a colocalization of both elements in some areas in the intestine of the animal with transfer colitis after Eu-VSOP injection, which can be explained by the MNP accumulation in these areas (Fig. 5C and D). This is consistent with the observations of Golusda *et al.* who were able to show that VSOP-loaded monocytes migrated into inflamed areas and endothelial cells took up VSOP.^23^ Eu hotspots with up to about 15 μg g^−1^ Eu were detected. The phosphorus image shows the shape of the intestine sample section for orientation (Fig. 5E). In contrast, the liver tissue sections show rather homogeneous distributions for Eu and Fe (Fig. 5A and B). The LA-ICP-MS results fit well with the MPS data (Fig. 6 and S2 in the SI) as the MPS signal amplitude *A*_3_ is also higher for the animals injected with Eu-VSOP.

#### 2.2.4 Correction of MPS quantification

Using the magnetic fingerprint, contaminations were identified and excluded from analysis in colon (5 out of 10), caecum (3 out of 10), and small intestine (5 out of 16) samples derived from healthy mice. In contrast, no contaminations were detected in the liver and spleen, where a high amount of Eu-VSOP was quantified.

Additionally, significant amounts of Eu-VSOP were measured in the colon, kidney, small intestine, and caecum, further confirming Eu-VSOP biodistribution. After excluding contaminated samples, none of the tissue samples from animal groups neither with intestinal inflammation nor the healthy one exhibited signals above the limit of detection (LOD) without Eu-VSOP injection. However, in Eu-VSOP-injected animals, liver and spleen tissue consistently showed high Eu-VSOP levels, independent of the mouse model (Fig. 6A–C).

Notably, colon tissue from mice with DSS-induced colitis exhibited a trend toward higher Eu-VSOP accumulation, suggesting a potential influence of intestinal inflammation on Eu-VSOP biodistribution.

## 3. Conclusions

In this study, Magnetic Particle Spectroscopy (MPS) was employed to detect and quantify Europium-doped Very Small Superparamagnetic Iron Oxide Particles (Eu-VSOP) in mouse models of intestinal inflammation. MPS is a highly sensitive technique that enables precise detection of superparamagnetic nanoparticles (MNPs) by measuring their nonlinear magnetic response when subjected to an alternating magnetic field. Our findings demonstrate that MPS signal amplitude scales linearly with MNP concentration. This linear relationship confirms MPS as a reliable and quantitative method for assessing MNP accumulation in biological samples. Beyond simple quantification, MPS enables the clear distinction between different MNP types, ensuring specificity in nanoparticle detection by additionally utilizing spectral shape and phase information. In contrast, neither ICP-OES or ICP-MS after digestion nor LA-ICP-MS allow a differentiation between MNP accumulation or contamination. Although this is possible using Eu-doped VSOP, it is not feasible in clinical routine due to the toxicity of Eu.

A key challenge identified in this study was the presence of magnetic contamination in food, which exhibited the same spectral characteristics as fecal samples from mice. Why is this an issue? The gastrointestinal tract is constantly exposed to external materials, including dietary components containing trace magnetic substances.

Such contamination can interfere with MNP biodistribution studies, potentially leading to false-positive signals. Hence, such contamination must be considered in nanoparticle biodistribution studies. One of the primary sources of unintended magnetic background signals is commercially available animal food, which can contain trace amounts of ferromagnetic particles due to manufacturing processes involving metallic machinery and/or natural mineral content in some food ingredients (*e.g.*, iron-rich components). Apart from diet, animal housing materials can also introduce magnetic artifacts into experimental data. Metal cages and feeding trays pose a significant risk, as animals may inadvertently ingest microscopic metal particles. Similar issues have been reported in another study, where magnetic contamination in commercial animal food and ingestion of metal cage components led to significant background signals during magnetic investigations of rabbits.^24^ To address this issue, previous research has recommended non-magnetic housing materials (*e.g.* Plexiglas) and a contamination-free diet (*e.g.*, fresh vegetables only).^25^ Similar challenges have been reported in MPI studies, where signals from metal shavings shed by surgical instruments, iron in animal feed, fecal matter, and recycled paper products led to unusable or confounded images.^26^

To ensure accurate MPS quantification of Eu-VSOP in intestinal samples, we developed a method for differentiating Eu-VSOP from magnetic contamination by analyzing concentration-independent MPS parameters such as the harmonic ratio (*A*_5_/*A*_3_) and phase (*ϕ*_3_). The harmonic ratio is the ratio of the 5th to the 3rd harmonic component in the MPS spectrum, while the phase shift (*ϕ*_3_) is a parameter independent of concentration, reflecting unique magnetic properties of MNPs. This method allows for the exclusion of contaminated samples, preventing misinterpretations of Eu-VSOP biodistribution improving the accuracy and reliability of MPS-based studies.

The findings of this study have broader implications beyond MPS and can improve MRI interpretation in nanoparticle research. Magnetic contaminants in food can cause signal cancellations in MRI, which may appear identical to actual Eu-VSOP signals. This could lead to false conclusions about nanoparticle distribution in the intestine. Quality control measures could be post-MRI by analysing tissue samples after MRI investigations or pre-experimental by conducting quality checks on *e.g.*, nutrition. By implementing these strategies, this study provides a robust approach to ensuring reliable nanoparticle imaging and biodistribution data in future MNP-based biomedical applications.

Nevertheless, a limitation of the present study is the absence of alternative diets as a variable in the study design. This restricts the ability to compare the effects of different dietary interventions and may limit the generalizability of our findings. Future research should consider including various dietary approaches to provide a more comprehensive understanding of their potential impacts on intestinal contamination.

In conclusion, this study highlights MPS as a powerful tool for both quantification and differentiation of MNPs, addressing a critical challenge of magnetic contamination in nanoparticle biodistribution studies. By introducing a method to separate Eu-VSOP signals from unwanted background interference, the findings contribute to more accurate imaging and analysis in both MPS and MRI-based studies.

## 4. Methods

### 4.1 Magnetic nanoparticles

Europium-doped very small superparamagnetic iron oxide particles (Eu-VSOP) were synthesized at Charité – Universitätsmedizin Berlin, Department of Radiology following the protocol by de Schellenberger *et al.*^27^ The resulting formulation had an iron concentration of *c*(Fe) = 0.5 mol L^−1^ with 13% citric acid (w/w total Fe), 3 g L^−1^ sodium glycerophosphate, 2 g L^−1^*N*-methylglucamine, and 60 g L^−1^ mannitol. The synthesis was adapted to produce the final pharmaceutical formulation of VSOP-C184, used in clinical trials.^28^ To enable clear analytical differentiation from endogenous iron or iron contaminations, VSOP were doped with europium (Eu) by substituting ferric ions with a 5% weight ratio of Eu^3+^ to Fe^3+^. This modification, resulting in Eu-VSOP, does not affect the magnetic properties of the particles.^29^ The Eu-VSOP exhibit small hydrodynamic sizes of *d* = 10(2) nm.^27^

Furthermore, we used two commercially available MNP systems, namely Ferucarbotran (marketed under the name Resovist® by Bayer Schering Pharma, GER) as well as Ferumoxides (marketed under the name Endorem® by Guerbet, FRA). Resovist® is an aqueous suspension of iron oxide nanoparticles coated with carboxydextran consisting of single and multi-core MNP with a hydrodynamic size of *d*_h_ = 61.6(9) nm. In contrast, Endorem® consists of several individual small iron oxide nanoparticles embedded in a dextran matrix that forms one larger particle with *d*_h_ = 11(4) nm. Additionally, a multicore particle system (MCP) recently developed at the Charité – Universitätsmedizin Berlin with *d*_h_ = 53(2) nm was included in our investigations.^30,31^

Chemicals and solvents used in this work were obtained from Sigma-Aldrich (GER).

### 4.2 Quantification of iron by magnetic particle spectroscopy

Magnetic Particle Spectroscopy (MPS) is based on detecting the nonlinear dynamic response of the magnetic moments of the MNP when exposed to an oscillating magnetic excitation field. Taking only the nonlinear components of the magnetization response into consideration enables us to exclude magnetic signal contributions from diamagnetic biological tissue and paramagnetic blood iron, which exhibit linear magnetization responses, only.

For measurements, a PCR tube with a maximum capacity of 140 μL was placed into the pick-up coil of a commercial MPS device (MPS-3, Bruker BioSpin, Ettlingen, GER) operating at a fixed frequency of *f*_ex_ = 25 kHz. The sample was subjected to a sinusoidal oscillating magnetic field with an excitation amplitude of *B*_ex_ = 25 mT. The system is capable of detecting dynamic magnetic moments down to 5 pA m^2^ with an outstanding dynamic range of six orders of magnitude, ensuring precise detection of the magnetization response. Signals at the fundamental excitation frequency (linear response and excitation signals) are effectively suppressed by high pass filtering together with gradiometric design of the receive coil.

The response of the MNP is recorded in the time-domain and then processed using Fourier transformation and averaging, yielding the characteristic MPS amplitude *A*_n_ and phase *ϕ*_n_ spectra, where the dominant response occurs at odd harmonics (*i.e.*, *n* = 3, 5, 7, *etc.*) of the excitation frequency *f*_ex_. The harmonic amplitudes *A*_n_ of the MPS spectrum are directly proportional to the amount of MNP (or amount of MNP iron), with the third harmonic amplitude *A*_3_ typically used for quantification since it exhibits the strongest signal contribution whereas the higher amplitudes monotonically decrease with increasing harmonic order *n*. First, the MPS spectrum of a reference sample of known iron amount *m*(Fe) is measured from which the specific moment 
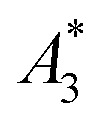
 (moment normalized to the iron amount in units A m^2^ kg^−1^ (Fe)) can be determined for each MNP system. Then, the iron amount of a tissue sample is obtained by dividing the measured *A*_3_ moment by 
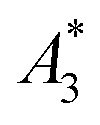
.

The shape of the amplitude spectrum *A*_n_ is independent of the MNP concentration, reflecting instead the intrinsic properties of the MNP determined by magnetic moment, magnetic anisotropy, and their interaction with the surrounding environment. This spectral shape is characterized by the ratio of the 5th to the 3rd harmonics (*A*_5_/*A*_3_). Structural and environmental changes of the MNP also influence the phase spectrum *ϕ*_n_, which remains largely independent of particle quantity but is highly sensitive to MNP properties.^32^ The phase *ϕ*_3_ is considered as a measure of the lag between oscillating excitation field and response of the magnetic moments of the MNP. Note, that in the used MPS device the phases are (arbitrarily) preceded by a negative sign.

The limit of detection (LOD) of the MPS was determined according to the guidance of the International Union of Pure and Applied Chemistry (IUPAC): LOD (*A*_n_) = *μ* + 3*σ*, where *μ* is the mean and *σ* the standard deviation of 20 empty-sample-holder measurements to determine the background level of the MPS device (LOD = 2 × 10^−11^ A m^2^).

### 4.3 Animals

All animals were approved by the regional Office for Health and Social Affairs in Berlin (Landesamt für Gesundheit und Soziales Berin, LAGeSo, G0422/17). Recombination activating gene 1-deficient mice (Rag1^tm1Mom^) on a C57BL/6 background were purchased from Jackson Laboratory (Bar Harbor, USA) and bred under specific pathogen free conditions at the Research Institute for Experimental Medicine (FEM; Charité – Universitätsmedizin Berlin). Recombination activating gene 1-deficient (RAG1 ko) mice produce no mature B and T cells. Inbred wild type C57BL/6 mice were purchased from Charles River, Germany. Animals were housed under conventional conditions at the animal facilities in Charité Campus Mitte of the Research Institute for Neurological Science (NWFZ). All animals were kept in polycarbonate cages and had free access to sterile standard chow and drinking water.

All experiments were performed in accordance with the German legislation on the protection of animals and approved by the local authorities (Landesamt für Gesundheit und Soziales, registration number G0422/17).

### 4.4 Colitis induction

Colitis was induced in 8–9 weeks old female C57BL/6 wildtype mice (WT) by providing 3% dextran sodium sulfate (DSS; MW 36 000–50 000, MP Biomedicals, Santa Ana, CA, USA) *via* the drinking water from day 0 to 6 followed by pure drinking water until day 8 or day 10 (full-blown DSS-induced colitis). Control animals constantly received pure drinking water.

Transfer colitis was induced in 8–9 weeks old female RAG1 ko mice by intraperitoneal (i.p.) injection of 4 × 10^5^ syngeneic CD4^+^CD45RB^hi^ T cells in 200 μL PBS (ThermoFisher Scientific). CD4^+^CD45RB^hi^ T cells were isolated from female wild type C57BL/6 mice according to Maschmeyer *et al.*^33^ Control animals received 200 μL PBS i.p.

Mice were controlled regularly for clinical signs of colitis and electively sacrificed when meeting humane endpoints. Stool samples were scored as described previously (PMID: 25596454). Histological scoring was done as described by Erben *et al.* (PMID: 25197329). Representative pictures of histopathological scoring are provided in Fig. S6.

### 4.5 Eu-VSOP application and tissue collection

Mice were anesthetized *via* a mask with 2% isoflurane. Eu-VSOP or PBS were applied intravenously *via* the tail vein. Control and colitic animals received 0.03 mmol Eu-VSOP per kg of body weight. 90 min after Eu-VSOP injection, the animals were sacrificed, and tissues were collected. Colon, small intestine, caecum, liver, spleen, and kidney were dissected with ceramic instruments free from any metal to avoid tissue contamination. Tissue samples were only in contact with instruments based on plastic or ceramic material. After dissection, tissue samples were rinsed with PBS. For the LA-ICP-MS measurements thin sections of formalin-fixed, paraffin-embedded intestine or liver samples with a thickness of 4 μm were prepared and mounted on SuperFrost Plus adhesion slides (Thermo Fisher Scientific, Schwerte, GER). Feces samples were taken from the colon and caecum. All samples were put in polypropylene tubes and stored at −80 °C until MPS measurement or sample digestion for the analysis with ICP-MS or ICP optical emission spectrometry (ICP-OES). Fortified (autoclaved and γ irradiated) complete feed for rats and mice (ssniff, Soest, Germany) was collected in polypropylene bags and stored at room temperature until MPS measurement or digestion.

### 4.6 Elemental mapping with LA-ICP-MS

For the elemental mapping a laser ablation system (NWR-213, Elemental Scientific Lasers, Bozeman, MT, USA) was coupled to an ICP time-of-flight mass spectrometer (ICP-TOFMS) from TOFWERK AG, Thun, Switzerland. Thin sections of formalin-fixed, paraffin-embedded intestine or liver samples (thickness 4 μm) from colitic animals with (animal #71) and without (animal #83) Eu-VSOP injection were measured. Eu was quantified using micro-droplets of spiked gelatin. For Fe and P the intensity distributions for ^31^P and ^56^Fe are shown. Additional details are given in the SI (S3).

### 4.7 Sample digestion and analysis by ICP-MS or ICP-OES

Feces caecum samples from mice with and without Eu-VSOP injection (each from 3 different animals) as well as parts of mouse feed pellets were digested in a high pressure asher (HPA-S, Anton Paar, Graz, Austria) with 1 mL concentrated nitric acid (purified by subboiling distillation) at 300 °C for 2 h at 100 bar using 15 mL quartz glass vessels. Blanks were prepared in a similar manner. The selected digestion parameters allowed a complete decomposition of the feces. A small residue remained after digestion of the feed samples, which was separated by centrifugation and filtration. The residue could be silicate material which cannot be completely decomposed with nitric acid. The digests were quantitatively transferred into 15 mL Falcon tubes and diluted with deionized water (18.2 MΩ cm, Milli-Q water purification system from Millipore, Eschborn, GER) for the subsequent measurements with ICP-MS or ICP-OES.

An ICP sector-field mass spectrometer (Element XR, Thermo Fisher Scientific, Bremen, GER) was used for the Eu quantification and an Arcos II ICP-OES (SPECTRO Analytical Instruments, Kleve, GER) for Fe due to the higher Fe concentrations in the digests. Additional details can be found in the SI (S4, S5).

### 4.8 Statistical analysis

For each group, descriptive statistics were calculated. The mean and the simple (unadjusted) standard deviation (SD) were determined. The standard deviation is reported as a measure of data dispersion and corresponds to a coverage interval of approximately 68% of the data, assuming a normal distribution. All calculations were performed using Origin software.

## Author contributions

Study conception and design, NL, LG, FW, AK; methodology, NL, LG, FW, DP, HT; data collection, NL, LG, HT, MS, JS, CF; formal analysis, NL, LG, HT, AK, DP; interpretation of results, NL, AK, HT, DP, FW; writing—original draft preparation, NL, LG; writing—review and editing, NL, LG, HT, AK, DP, FW; visualization, NL, HT, LG; supervision FW, AK, MT; funding acquisition, AK, FW, MT, HT. All authors have read and agreed to the published version of the manuscript.

## Conflicts of interest

The authors declare no conflict of interest.

## Supplementary Material

NA-007-D5NA00452G-s001

## Data Availability

The data supporting this article have been included as part of the SI. Supplementary information is available. See DOI: https://doi.org/10.1039/d5na00452g.
