# Supplementary material for: Magnetic particle spectroscopy for Eu-VSOP quantification in intestinal inflammation: distinguishing nanoparticle signals from dietary contamination
Source: Nanoscale Adv. 2025 Aug 28;7(20):6525–34. doi: 10.1039/d5na00452g (PMC12406948; doi:10.1039/d5na00452g)
Supplement: NA-007-D5NA00452G-s001 [file NA-007-D5NA00452G-s001.pdf]

ARTICLE

Supplementary Material - Magnetic Particle Spectroscopy for Eu-VSOP Quantification in Intestinal Inflammation: Distinguishing Nanoparticle Signals from Dietary Contamination

Received 00th January 20xx,
Accepted 00th January 20xx

DOI: 10.1039/x0xx00000x

Norbert Löwa[a], Laura Golusda[b,c], Daniela Paclik[b], Heike Traub[d], Mathias Schannor[d], Jessica Saatz[d], Christian Freise[e], Matthias Taupitz[f], Britta Siegmund[b], Anja A. Kühl[c], Frank Wiekhorst[a]

Magnetic nanoparticles are gaining increasing attention as a promising alternative to gadolinium-based contrast agents in magnetic resonance imaging, primarily due to their low toxicity. In this study, we investigated the use of magnetic iron oxide nanoparticles in mouse models of intestinal inflammation to assess their potential for detecting changes in the extracellular matrix. For magnetic quantification, we employed Magnetic Particle Spectroscopy, which offers high sensitivity and minimal interference from biological tissue. However, we observed significant variations in magnetic signals within the intestine, as well as measurable signals in control animals, indicating possible magnetic contamination. By doping the nanoparticles with europium, we were able to confirm this suspicion through quantitative elemental analysis. Examination of mouse feed and feces allowed us to identify the source of contamination. Based on these findings, we developed a method to reliably distinguish genuine signals of magnetic nanoparticles from those caused by external magnetic contaminations. This approach is essential to ensure reliable results in future diagnostic and preclinical research.

S.1 Influence of sample dilution on MPS signals

Figure S1 presents the MPS results for the Eu-VSOP sample as a function of iron mass (m(Fe)), divided into three panels displaying the harmonic amplitude, harmonic ratio, and phase response for varying Eu-VSOP quantities.

Harmonic amplitude: The third harmonic amplitude (A_3) as a function of the iron mass (m(Fe)) (Fig. S1A) exhibits a clear increasing trend. A linear fit to the data (red line, slope A_3* = 0.600(5) Am^2/kg) confirms that the MPS signal scales linearly with iron mass within the measured range. The horizontal dashed line represents the LOD, demonstrating that all measurements above approximately 30 ng of iron are well above the detection threshold. This highlights the high sensitivity of MPS in detecting even small Eu-VSOP quantities.

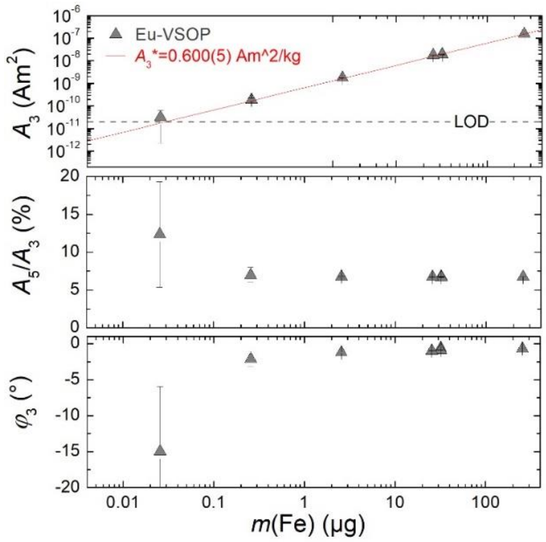

Figure S1: Characteristic MPS signals of a serial dilution of Eu-VSOP. While A_3* (A) scales linearly with MNP iron content, the harmonic ratio (A_5/A_3) (B) and the phase of the third harmonic (ϕ_3) (C) remain stable down to the detection limit (LOD).

The observed linearity indicates a direct proportionality between the number of magnetic nanoparticles and the third harmonic response - a fundamental characteristic of MPS that enables quantitative nanoparticle concentration analysis. The consistency of this trend across the measured iron mass range underscores the suitability of MPS for highly sensitive nanoparticle detection.

Harmonic Ratio: Figure S1B illustrates the ratio of the fifth to the third harmonic amplitudes (A_5/A_3) as a function of iron mass, serving as a key indicator of the dynamic magnetic

[a.] *Physikalisch-Technische Bundesanstalt (PTB), Working Group 8.23 Metrology for Magnetic Nanoparticles, Abbestr. 2-12, 10587 Berlin, Germany*
[b.] *Charité– Universitätsmedizin Berlin, corporate member of Freie Universität Berlin and Humboldt- Universität zu Berlin, Department of Gastroenterology, Infectious Diseases and Rheumatology, Campus Benjamin Franklin, Hindenburgdamm 30, 12200 Berlin, Germany*
[c.] *Charité - Universitätsmedizin Berlin, corporate member of Freie Universität Berlin and Humboldt- Universität zu Berlin, iPATH.Berlin, Campus Benjamin Franklin, Hindenburgdamm 30, 12200 Berlin, Germany*
[d.] *Bundesanstalt für Materialforschung und -prüfung (BAM), Division 1.1 Inorganic Trace Analysis, Richard-Willstätter-Str. 11, 12489 Berlin, Germany*
[e.] *Charité – Universitätsmedizin Berlin, corporate member of Freie Universität Berlin and Humboldt- Universität zu Berlin, Department of Radiology-Experimental Radiology, Campus Mitte, Virchowweg 11, 10117 Berlin, Germany*
[f.] *Charité – Universitätsmedizin Berlin, corporate member of Freie Universität Berlin and Humboldt- Universität zu Berlin, Department of Radiology, Campus Benjamin Franklin, Hindenburgdamm 30, 12200 Berlin, Germany*
† Electronic supplementary information (ESI) available. See DOI: 10.1039/x0xx00000x

Please do not adjust margins

properties of the particles, including relaxation dynamics and magnetic anisotropy. For the Eu-VSOP sample, this ratio remains relatively low and stable across most iron mass values, except at the lowest concentrations, where a notable increase is observed.

This increase at very low Eu-VSOP masses suggest stronger contributions from higher-order harmonics, potentially due to factors such as a limited number of particles, environmental interactions, or measurement noise. However, for masses exceeding 200 ng, the harmonic ratio stabilizes at approximately 5-10%, indicating a consistent magnetic response and stable relaxation dynamics at higher concentrations.

Phase angle: Figure S1C presents the phase angle of the third harmonic (ϕ_3) as a function of iron mass. The phase angle remains consistently around -10°, with increased measurement uncertainty observed below 200 ng, likely due to weaker signal intensity and proximity to the detection limit.

The stability of the phase angle at higher iron masses suggests that the magnetic relaxation dynamics of the Eu-VSOP sample remain consistent as the concentration increases, reinforcing the idea that MPS can reliably characterize magnetic nanoparticle properties across a wide range of concentrations.

This highlights the sensitivity of MPS for detecting and characterizing Eu-VSOP nanoparticles across a wide range of iron concentrations. The linear increase in (A_3) with iron mass (m(Fe)), coupled with the relatively stable harmonic ratio (A_5/A_3) and phase angle (ϕ_3) at higher concentrations, confirms that MPS provides a robust fingerprint for the magnetic response of Eu-VSOP.

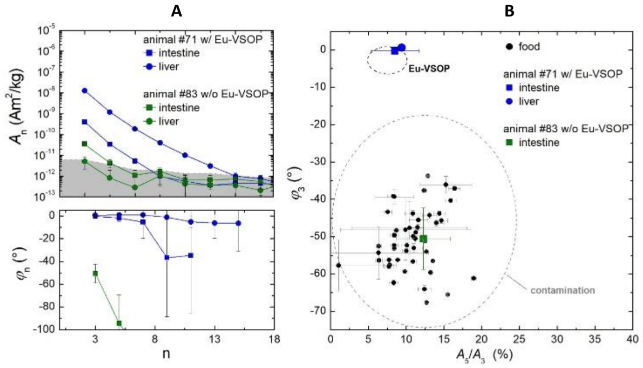

Figure S2: The results of the MPS measurements of tissue samples (liver: circles; spleen: squares) from animal with Eu-VSOP injection (blue) and control animal without Eu-VSOP injection (green) are shown as amplitude (A_n) and phase spectrum (ϕ_n). The noise floor is indicated by a gray-shaded area (A). A two-dimensional plot of the phase (ϕ_3) against the amplitude ratio (A_5/A_3) allows a clear distinction between contaminated signals (lower circle) and Eu-VSOP signals (upper circle) (B).

S2. Magnetic Particle Spectroscopy on tissue samples for LA-ICP-MS

The samples analyzed using LA-ICP-MS were subsequently measured by MPS. The amplitude spectrum (A_n) of the liver and intestine from the animal with Eu-VSOP injection shows elevated signals well above the noise floor (Fig. S2a) and a phase (ϕ_n) near 0°, consistent with Eu-VSOP characteristics. Interestingly, the amplitude spectrum (A_n) of the intestine from the animal without Eu-VSOP injection also exhibits a signal above the noise floor (Fig. S2A, grey shaded area), whereas the signal from the liver, which typically accumulates foreign substances due to its role in the mononuclear phagocyte system, remains below the noise floor. The third harmonic phase (ϕ_3) of this intestinal sample is around 50°, which, in combination with the harmonic ratio (A_5/A_3), enables its classification as contamination (Fig. S2B).

S3. Elemental mapping with LA ICP time-of-flight mass spectrometry (LA-ICP-TOFMS)

An NWR-213 laser ablation system (Elemental Scientific Lasers, Bozeman, MT, USA) equipped with a Two-volume sample chamber was coupled to an ICP time-of-flight mass spectrometer (ICP-TOFMS; icpTOF 2R from TOFWERK AG, Thun, Switzerland). Instrumental settings were optimized daily using NIST SRM 612 (Trace Elements in Glass; National Institute for Standards and Technology, Gaithersburg, MD, USA) focussing on high intensities and low oxide formation rates. Helium was used as carrier gas and argon (0.7 L min^{-1}) was added via a Y-piece before reaching the ICP torch. The ICP-TOFMS was used in no gas mode and with a dwell time of 50 ms. The LA operating parameters are summarised in Table S3. Tissue thin sections, mounted on microscopic glass slides (SuperFrost Plus adhesion slides, Thermo Fisher Scientific, Schwerte, GER), were inserted into the sample chamber and ablated line by line. For the elemental mapping a special imaging mode, named "differential scanning" mode, was used[1,2]. The laser energy was optimized to ablate the sample completely with every laser shot without affecting neighbouring regions. Scan rate and repetition rate were adjusted so that the laser spots are overlapping. Thus, the signal of the sample is generated only by the new incremental area ablated with each laser shot. Therefore, the resolution in scan direction (x) is better than the laser spot diameter. The resolution in y direction is given by the distance between the line scans. The pixel size given for the elemental maps indicates the spatial resolution in x and y direction in µm.

Eu was quantified using an external calibration based on micro-droplets of Eu doped gelatine following recently established procedures[3–5]. In brief, gelatin from cold water fish skin (Sigma-Aldrich, Taufkirchen, GER) was spiked with various amounts of a europium standard solution (Eu Certipur standard for ICP, 1 g/L, Merck Millipore, Darmstadt, GER). For the spotting process a micro array spotter (sciFLEXARRAYER S3, Scienion AG, Berlin, GER) equipped with a piezo dispense capillary (PDC 70, coating type 3) was employed. Micro-droplets of spiked gelatine solution (gelatin 1 % w/w) with a droplet volume of about 300 pL were deposited on a glass slide (Superfrost Plus, Thermo Scientific, Schwerte, GER). The sum of the Eu signal intensities and the absolute Eu masses within the gelatin droplets were used to set up the calibration curve. To obtain element contents for each pixel of the tissue samples, the total ablated tissue mass per pixel was calculated based on the tissue thickness (4 µm), the tissue density, and the ablated area per pixel.

For the data post processing the HDF5 files from the ICP-TOFMS software were converted into txt files. Afterwards the software

 This journal is © The Royal Society of Chemistry 20xx

Please do not adjust margins

tool MassImager, version 3.63b (developed by Robin Schmid, University of Münster, GER) was used to create the color-coded elemental maps for Eu, Fe and P using the raw data of the isotopes ^{31}P, ^{56}Fe and ^{151}Eu, respectively. For Eu quantitative elemental maps are shown and the intensity distributions for ^{31}P and ^{56}Fe.

Table S3: LA operating parameters

Laser	Nd:YAG at 213 nm
Measurement mode	Imaging
He chamber gas flow / mL min^{-1}	1000
Laser spot size / µm	50 (intestine), 100 (liver)
Scan rate / µm s^{-1}	50 (intestine), 100 (liver)
Repetition rate / Hz	20
Fluence / J cm^{-2}	0.7
Line spacing	40 (intestine), 80 (liver)

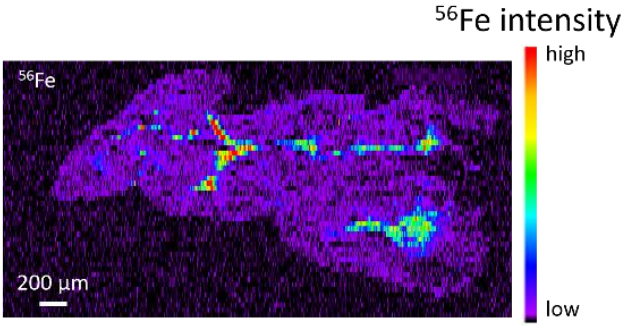

Figure S3: Fe distribution of an intestine thin section from a control animal without Eu-VSOP injection (animal #83) measured with LA-ICP-TOFMS. Pixel size 2.5 µm x 40 µm, scale bar 200 µm.

S4. ICP-MS measurements

The ICP sector-field mass spectrometer Element XR (Thermo Fisher Scientific, Bremen, GER) was operated under standard conditions in medium mass resolution and tuned with a multielement solution for sensitivity and repeatability. The operating parameters are given in Table S4.

Table S4: Element XR experimental parameters

Sample introduction system	Cyclonic spray camber (glass), MicroMist nebulizer (glass, 200 µL/min, self-aspirating)
Sample and skimmer cone	Ni
Mass resolution	Medium (*R* = 4000)
Measured isotopes	^{56}Fe, ^{151}Eu, ^{153}Eu, ^{175}Lu (internal standard)

For the calibration europium standard solutions prepared from a stock solution (Eu Certipur standard for ICP, 1 g/L, Merck Millipore, Darmstadt, GER) were used. Lutetium (Lu Certipur standard for ICP, 1 g/L, Merck Millipore) was used as internal standard. All dilutions were made with deionized water (18.2 MΩ cm, Milli-Q water purification system from Millipore, Eschborn, GER). The mean values of the intensities of the isotopes ^{151}Eu and ^{153}Eu were used for the data evaluation.

S5. ICP-OES measurements

Standard measurement conditions for solution analysis with an ICP-OES Arcos II (SPECTRO Analytical Instruments, Kleve, GER) were used for iron determination. The instrument was equipped with a Scott spray chamber (glass) and a cross-flow nebulizer.

Iron standard solutions prepared from a stock solution (Fe Certipur standard for ICP, 1 g/L, Merck Millipore, Darmstadt, GER) were used for calibration. All dilutions were made with deionized water (18.2 MΩ cm, Milli-Q system from Millipore, Eschborn, GER). The mean values of the measured values for the Fe wavelengths 238.204 nm, 239.562 nm and 259.941 nm were used for the data evaluation.

S6. Histopathological manifestations of colitis models in colon sections

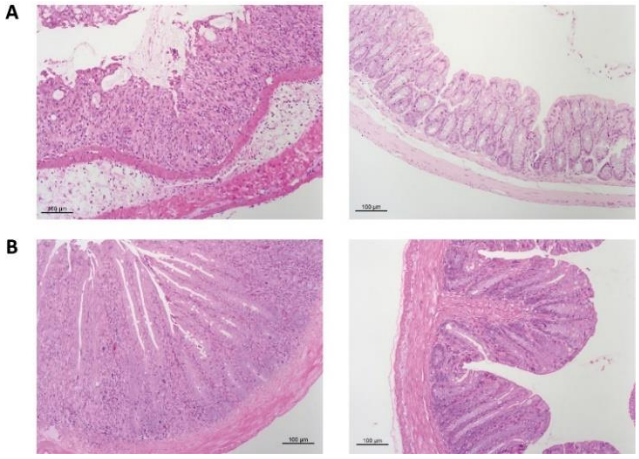

Figure S6: Representative images of H&E-stained colon sections of DSS-induced colitis with manifestation of severe ulceration and tissue changes (A left), transfer colitis with severe crypt hyperplasia and immune cell infiltration (B left) and respective healthy control (A + B right).

References

1 D. Drescher, C. Giesen, H. Traub, U. Panne, J. Kneipp and N. Jakubowski, Quantitative imaging of gold and silver nanoparticles in single eukaryotic cells by laser ablation ICP-MS, *Anal. Chem.*, 2012, **84**, 9684–9688.

2 L. Mueller, H. Traub, N. Jakubowski, D. Drescher, V. I. Baranov and J. Kneipp, Trends in single-cell analysis by use of ICP-MS, *Anal. Bioanal. Chem.*, 2014, **406**, 6963–6977.

3 A. Schweikert, S. Theiner, D. Wernitznig, A. Schoeberl, M. Schaier, S. Neumayer, B. K. Keppler and G. Koellensperger, Micro-droplet-based calibration for quantitative elemental bioimaging by LA-ICPMS, *Anal. Bioanal. Chem.*, 2022, **414**, 485–495.

4 A. Schweikert, S. Theiner, M. Šala, P. Vician, W. Berger, B. K. Keppler and G. Koellensperger, Quantification in bioimaging by LA-ICPMS - Evaluation of isotope dilution and standard addition enabled by micro-droplets, *Anal. Chim. Acta*, 2022, **1223**, 340200.

5 S. Theiner, E. Foels and G. Koellensperger, Advancing elemental analysis by collision/reaction cell technology and micro-droplet calibration for bioimaging applications by LA-ICP-TOFMS, *Anal. Chim. Acta*, 2024, **1332**, 343345.
